# Use of Schirmer strips and conjunctival swabs for virus detection on the ocular surface of adults: a scoping review

**DOI:** 10.5935/0004-2749.20230032

**Published:** 2023

**Authors:** Luís Expedito Sabage, Alessandra Mazzo, Josmar Sabage, Taylor Endrigo Toscano Olivo, Carlos Ferreira Santos, Luiz Fernando Manzoni Lourençone

**Affiliations:** 1 Faculdade de Odontologia de Bauru, Universidade de São Paulo, Bauru, SP, Brazil; 2 Hospital de Reabilitação de Anomalias Craniofaciais, Universidade de São Paulo, Bauru, SP, Brazil; 3 Instituto Lauro de Souza Lima, Bauru, SP, Brazil

**Keywords:** Antigen, surface/isolation & purification, Conjunctiva, Tears, Eye protein/analysis, Specimen handling, Polymerase chain reaction/methods, COVID-19, Eye manifestations, Antígeno de superfície/isolamento & purificação, Túnica conjuntiva, Lágrimas, Proteína do olho/análise, Manejo de espécimes, Reação em cadeia da polimerase/métodos, COVID-19, Manifestações oculares

## Abstract

Schirmer strips and conjunctival swabs are used in ophthalmology for the collection of tears and fluids. One of the biggest challenges during the COVID-19 pandemic has been accurate diagnosis and, in some cases, ocular manifestations are among the first symptoms. In this context, this study aimed to collect evidence to support the use of Schirmer strips and conjunctival swabs as a method of sample collection for viral analysis. A literature search was conducted following the Scoping Review protocol defined by The Joanna Briggs Institute. Studies were analyzed regarding virus research, collection methods, and sample analysis. The findings support that viruses can be detected on the ocular surface through analysis of Schirmer strips and conjunctival swabs. However, additional studies with larger samples and time data are necessary to confirm these conclusions.

## Abstract (pt)

A fita de Schirmer e o swab conjunctival são utilizados na oftalmologia como métodos de coleta para lágrimas e fluidos. Durante a pandemia da COVID-19, um dos desafios foi o diagnóstico correto e se sabe que, em alguns casos, as manifestações oculares podem ser um dos primeiros sintomas. Nesse contexto, este estudo tem como objetivo levantar evidência que destaque o uso de fitas de Schirmer e de swabs conjuntivais como método de coleta para análise viral. Conduziu-se uma revisão de literatura seguindo o protocolo para *Scoping Review* definido pelo *Joanna Briggs Institute*. Os pesquisadores analisaram os estudos em busca do vírus pesquisado, os métodos de coleta e os métodos de análise. Vírus podem ser detectados na superfície ocular através da análise de fitas de Schirmer e de swabs conjuntivais, entretanto novos estudos com populações maiores e com definições claras de tempo são necessários para conclusões mais assertivas no tema.

## INTRODUCTION

Viruses are intracellular parasites that are the smallest known infectious agents. Although the mechanisms of viral disease are still not completely clear, there are several factors that directly contribute to viral tropism, such as viral receptors in the host cell, the specific cell line, and physical barriers that enable and/or inhibit infections. Once inside a cell, the virus may damage or destroy it through direct cytopathic effects, host antiviral immune responses, and/or transformations of the infected cells^(1)^.

The eye is a known site of viral infections, and viruses may appear in the intra- or extraocular space without visible systemic reverberation and affect multiple structures with variable manifestations^(2,3)^. Viral diagnosis is made based on clinical signs/symptoms and laboratory test results. Various laboratory tests are available with the specificity and sensitivity varying from one microorganism to another. Cell culture and analysis of genetic material from samples collected from blood, mucosa, or secretions are the main methods.

Based on clinical information and previous experience, the physician should decide between available diagnoses considering the patient’s singularities. For the collection of samples from the eye, the most commonly used method is conjunctival swabs, although Schirmer strips have also shown good results.

### Current scenario

Ever since the World Health Organization (WHO) declared COVID-19 a global pandemic, the academic community has concentrated its efforts to treat the pandemic and help people return to normal life. The Chinese and ophthalmologist Li Wenliang, MD, was the first to report on the novel catastrophic virus SARS-CoV-2. It is now known that ocular manifestations can be one of the first symptoms of COVID-19 and, consequently, the eye may contribute to the understanding of COVID-19 pathophysiology^(4,5)^. Although several studies on ocular manifestations of SARS-CoV-2 have been conducted, the virus collection methods and associations with the ocular surface were not clearly stated.

### Schirmer test

The idea of collecting tears as a clinical test was first introduced in 1900 by Köster^(6)^. The test consists of the placement of filter paper on all extensions of the conjunctival sac while the nasal mucosa ies stimulated to produce tearing caused by nasal irritation. The objective of the test is to exhaust tear production to evaluate the function of lacrimal glands. As this test can take up to 90 min to perform, it is not viable for daily medical practice^(6)^.

In 1903, Otto Schirmer, a German ophthalmologist, shortened the length of the paper strips and quantified tear production for 5 min using three methods, each of which analyzed a distinct tearing stimulation pathway: ocular and palpebral mucosa, nasal mucosa, and the retina^(6,7)^. In the last century, several modifications have been made to the Schirmer test. Nevertheless, this test remains important in the quantification and standardization of tear volume. Currently, a filter paper strip measuring 60-mm long and 5-mm wide is inserted at the temporal side of the conjunctival sac while the patient’s eyes are closed. The strips are removed after 5 min and the wet part is measured. A result greater than 15 mm is considered to be normal, but the value varies with medication use, age, and the presence of chronic diseases^(8,9)^.

### Conjunctival swabs

Conjunctival swabs are the most commonly used method for microbiological analysis because they allow the collection of cells and materials spread in the conjunctival sac instead of exclusively tears. One exception is in-office rapid antigen tests for adenovirus (AdV) because they are faster and present a good sensitivity and specificity of 89% and 94%, respectively(^10)^. The method of collection is fast and simple: a swab with a cotton tip is gently passed using rotational movements on the conjunctival sac^(11)^. Topical anesthesia can be used to make the procedure more comfortable for the patient, since there is no significant difference in the final results when samples are analyzed by polymerase chain reaction (PCR) methods^(12)^. Proxymetacaine 0.5% is recommended for ocular surface anesthesia since it shows the fewest bactericidal effects among commercially available eye drops^(13)^. It is also recommended to use sterile swabs, since calcium-containing swabs can inhibit polymerase activity^(14)^.

In this context, this study aims to collect and evaluate scientific evidence of the use of Schirmer strips and conjunctival swabs as a method of virus collection on the ocular surface.

## METHODS

A literature review was conducted according to The Joanna Briggs Institute (JBI) recommendations for scoping reviews^(15)^. All searches and publication access were completed in June 2020 and there was no restriction on article publication date. The guiding research question “Is it possible to detect a virus on the ocular surface with the Schirmer test and/or conjunctival swab?” was formed for the selection and search of the studies. This was built through the Population, Concept, and Context strategy. In this way, “P” was defined as adult patients (>18 years old), “C” as the Schirmer test and conjunctival swab, and the last “C” as all viruses.

For the literature search, the following descriptors, synonyms, and keywords were used: “adult patients,” “Schirmer test,” “conjunctival swab,” and “virus.” The Boolean operators AND, NOT, and OR were used between descriptors. The controlled descriptors were “adult patient(s),” “Schirmer test,” “conjunctival swab(s),” and “virus.” The uncontrolled descriptors were “adult(s)” OR “patient(s),” “Schirmer strip(s),” and “ocular virus” OR “viral infection.” The search was performed using the databases PubMed, Web of Science, and *Blibioteca Virtual em Saúde* (BVS). Included articles were only those written in English, published in indexed sources, and with quantitative or qualitative approaches, primary studies, and reviews.

Insightful reading of the title, abstract, and keywords was performed to select the articles according to the previously established inclusion and exclusion criteria. When the title, abstract, and keywords were insufficient, the full text was also analyzed. All articles were called studies, enumerated in chronological order, and evaluated by three different researchers. Recommendations by JBI were adapted for the study singularities and used for data extraction. This article followed the Preferred Reporting Items for Systematic reviews and Meta-Analyses extension for Scoping Reviews (PRISMA-ScR) guidelines and checklist developed under EQUADOR (Enhancing the QUAlity and Transparency Of health Research) network guidance^(16)^.

## RESULTS

**Figure 1:**
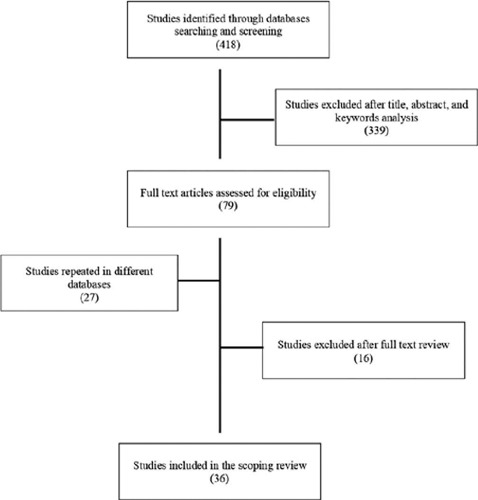
Summary of the systematic searches and processing of identifed studies.

Following the database search, 418 potential studies were identified. After reading the title, abstract, and keywords, 79 studies were selected. Of these, 27 articles were excluded because they were duplicate results. The full texts of the 52 remaining articles were read, and 16 were excluded for not answering the guiding question. Using the described methodology, the literature search identified 36 articles that met all criteria. This process is shown in figure 1 and the included studies are presented in table 1.

**Table 1:** Identifcation and details of the included studies

Nº	Year	Country	Purpose	Methodology
1^(17)^	1997	Japan	Compare IC and EIA tests for AdV detection	Experimental Quantitative Study
2^(18)^	1997	Germany	Evaluate type-specific primers for AdV	Experimental Quantitative Study
3^(19)^	1998	India	Develop and evaluate nested PCR as a tool for detecting AdV from conjunctival swabs	Descriptive Observational Study
4^(48)^	1999	USA	Determine the genetic stability of EBO-Z, and whether additional strains of EBO virus were circulating during Kikwit outbreak	Experimental Quantitative Study
5^(47)^	2000	China	Describe the application of conjunctival swab with PCR and virus culture to confirm the diagnosis of CMV retinitis in AIDS patients	Prospective Longitudinal Quantitative Study
6^(20)^	2000	UK	Develop a multiplex PCR for the detection of AdV, HSV, and Chlamydia trachomatis in conjunctival swabs	Experimental Quantitative Study
7^(21)^	2001	Taiwan	Evaluate the sensitivity and applicability of PCR and RT-PCR diagnoses for keratoconjunctivitis associated with viral infection	Experimental Quantitative Study
8^(30)^	2001	Netherlands	Develop a longitudinal analysis of VZV DNA on the ocular surface of patients with herpes zoster ophthalmicus	Experimental Quantitative Study
9^(31)^	2002	UK	Determine whether ocular shedding of EBV in the tear film is peculiar to patients with Sjogren’s syndrome, and whether coinfection with EBV occurs in the tear film	Experimental Quantitative Study
10^(22)^	2002	Austria	Investigate a rapid and sensitive PCR-based assay for the detection of adenoviral infections	Experimental Quantitative Study
11^(32)^	2002	France	Use a multiplex PCR to detect herpes viruses in tears from normal subjects and from patients with pathological conditions	Descriptive Quantitative Observational Study
12^(52)^	2004	Singapore	Determine the prevalence of virus in bodily excretions, and time of seroconversion in discharged patients with SARS	Experimental Quantitative Study
13^(23)^	2004	Brazil	Develop a rapid protocol to detect AdV in eye swab	Descriptive Quantitative Observational Study
14^(24)^	2005	UK	Determine if AdV persists on the ocular surface following adenoviral conjunctivitis	Experimental Quantitative Study
15^(25)^	2007	Japan	Establish a method of quantitative detection and rapid identification of AdV	Observational Quantitative Study
16^(26)^	2010	China	Test if high-density resequencing microarray can be applied to detection of viruses in conjunctival swabs for patients with conjunctivitis	Experimental Quantitative Study
17^(33)^	2011	Japan	Investigate if ICP0 of HSV-1 is detectable in the tear fluid of patients with HEK	Observational Quantitative Study
18^(34)^	2013	Korea	Analyze the methodological efficacy of the PCR assay for HSV-1 detection in tears	Experimental Quantitative Study
19^(27)^	2013	UK	Validate and introduce a simple boil extraction on dry swabs followed by amplification and realtime detection using “in-house” assays for HSV and AdV with RNaseP as an internal control	Experimental Quantitative Study
20^(35)^	2014	Spain	Evaluate the usefulness of PCR as a rapid diagnostic method compared with the viral culture, and to assess if conjunctival swabs samples were equivalent to corneal scrapings to diagnose of HK	Experimental Quantitative Study
21^(28)^	2015	UK	Estimate the diagnostic accuracy of the AdenoPlus point-of-care AdV test compared to PCR	Prospective Diagnostic Accuracy Study
22^(36)^	2016	Japan	Investigate diagnostic efficacy of PCR and ELISA for HSV in tears	Nonrandomized Prospective Cross-Sectional Study
23^(29)^	2016	India	Identify and characterize the viral etiological agents associated with keratoconjunctivitis	Retrospective Observational Study
24^(49)^	2016	UK	Report EBO virus RT-PCR data for body site and fluid samples from a large cohort of EBO virus survivors at clinic follow-up	Cross-Sectional Observational Study
25^(51)^	2017	Singapore	Check if Zika virus could be detected in human tears after the first week of infection	Descriptive Observational Study
26^(50)^	2018	Germany	Evaluate a non-invasive detection method for HPV in ophthalmic pterygia	Observational Prospective Case Control Study
27^(37)^	2019	UK	Investigate the use of a corneal impression membrane for the detection of HSV-1	Experimental Quantitative Study
28^(39)^	2020	China	Report the ocular characteristics and the presence of viral RNA of SARS-CoV- 2 in conjunctival swab specimens in a patient with confirmed Covid-19	Prospective Observational Case Study
29^(38)^	2020	China	Detect SARS-CoV-2 in eye sample of one Covid-19 patient with obstruction of common lacrimal ducts	Prospective Observational Study
30^(40)^	2020	India	Detect the presence of viral RNA of SARS-CoV-2 in conjunctival swab specimens of Covid-19 patients	Experimental Quantitative Study
31^(41)^	2020	China	Describe the clinical spectrum of ocular symptoms and laboratory test in conjunctival swab samples	Cross-Sectional Study
32^(42)^	2020	Canada	Present a case of Covid-19 with an initial medical presentation of keratoconjunctivitis	Descriptive Observational Case Study
33^(44)^	2020	USA	Understand evidence about SARS-CoV-2 and ocular infection	Narrative Review
34^(45)^	2020	China	Detect SARS-CoV-2 RNA in conjunctival swabs	Retrospective Observational Study
35^(46)^	2020	France	Describe the multiplicity of ocular manifestations of Covid-19 patients	Descriptive Observational Study
36^(43)^	2021	Spain	Evaluate the presence SARS-CoV-2 RNA in conjunctival swabs of Covid-19 patients	Cross-Sectional Study

IC: immunochromatography; EIA: enzyme immunoassay; EBO: Ebola virus; EBO-Z: EBO subtype Z; ICP0: infected cell protein 0; HEK: herpetic epithelial keratitis; HK: herpetic keratitis; ELISA: enzyme-linked immunosorbent assay

The three researchers analyzed all identified studies about virus research, collection methods, and sample analysis. Most of them were experimental or observational studies developed in the United Kingdom (7/36). Studies on the topic began in 1997, but 55.6% were only published in the years 2011-2021.

Most studies were on AdV^(17,18,19,20,21,22,23,24,25,26,27,28,29)^ and Hesper Simplex Virus (HSV)^(20,24,27,30,31,32,33,34,35,36,37,38)^, followed by SARS-CoV-2^(38,39,40,41,42,43,44,45,46)^. Varicella-Zoster virus (VZV) ^(30,31,32)^, cytomegalovirus (CMV)^(31,32,47)^, Epstein-Barr virus (EBV)^(31,32)^, Ebola (EBO)^(48,49)^, human papillomavirus (HPV)^(50)^, and Zika virus^(51)^ have also been identified on the ocular surface. Enterovirus (EV), coxsackievirus A24 variant (CA24v), and SARS-CoV were not positive on ocular samples collected using conjunctival swabs or Schirmer strips^(19,21,52)^.

The preferred collection method was conjunctival swab alone in 29 out of 36 articles; six studies used Schirmer strips and only one study compared both collection methods^(24)^. One study was a narrative review about SARS-CoV-2^(44)^. The majority of the samples were analyzed by PCR or reverse transcriptase-PCR (RT-PCR), with the exception of studies number 1^(17)^ and 17^(33)^ that used immunochromatography (IC) and enzyme immunoassay (EIA) and immunoblot analysis, respectively. Details of the methods and findings of the included studies are provided in table 2.

**Table 2:** Methodological details and main fndings of the included studies

Nº	Virus	Method of collection	Method of analysis	Nº of Patients recruited	Nº of Samples	Result (+ or −)
1^(17)^	AdV	CS	IC and EIA	130	130	+
2^(18)^	AdV	CS	PCR	68	68	+
3^(19)^	AdV/EV70/CA24v	CS	Direct smear, PCR and virus isolation	20	20	+/−/−
4^(48)^	EBO	CS	RT-PCR	7	38	+
5^(47)^	CMV	CS	Immunofluorescence and PCR	13	60	+
6^(20)^	AdV/HSV	CS	PCR	541	805	+/+
7^(21)^	AdV/EV70/CA24v	CS	PCR, RT-PCR, culture isolation, and neutralization test	113	113	+/−/−
8^(30)^	VZV, HSV	CS	PCR	21	246	+/−
9^(31)^	EBV-1/EBV-2/CMV/VZV/HSV	ST	PCR	54	54	+/+/−/−/−
10^(22)^	AdV	CS	PCR	15	15	+
11^(32)^	HSV-1/HSV-2/VZV/CMV/ EBV/HHV-6	ST	PCR	93	186	+/+/+/+/+/+
12^(52)^	SARS-CoV	CS	PCR	64	126	−
13^(23)^	AdV	CS	PCR	7	7	+
14^(24)^	AdV/HSV	ST and CS	PCR	30	90	+/−
15^(25)^	AdV	CS	PCR	133	133	+
16^(26)^	AdV	CS	High-density resequencing microarray and PCR	38	114	+
17^(33)^	HSV-1	ST	Immunoblot analysis	18	18	+
18^(34)^	HSV	ST	PCR	115	115	+
19^(27)^	HSV-1/HSV-2/AdV	CS	PCR	541	541	+/+/+
20^(35)^	HSV	CS	PCR	188	188	+
21^(28)^	AdV	CS	PCR and point-of-care test	109	109	+
22^(36)^	HSV	ST	PCR and ELISA	82	82	+
23^(29)^	AdV/EV	CS	PCR and RT-PCR	23	23	+/−
24^(49)^	EBO	CS	RT-PCR	112	92	−
25^(51)^	Zika virus	CS	RT-PCR	29	58	+
26^(50)^	HPV	CS	PCR	21	42	+
27^(37)^	HSV-1	CS	PCR	110	220	+
28^(39)^	SARS-CoV-2	CS	RT-PCR	1	8	+
29^(38)^	SARS-CoV-2/HSV-1 HHV-6B	CS	RT-PCR and PCR	1	20	+/+/+
30^(40)^	SARS-CoV-2	CS	RT-PCR	45	45	+
31^(42)^	SARS-CoV-2	CS	RT-PCR	2	4	+
32^(42)^	SARS-CoV-2	CS	RT-PCR	1	2	+
33^(44)^	SARS-CoV-2			Narrative Review		
34^(45)^	SARS-CoV-2	CS	RT-PCR	33	66	+
36^(46)^	SARS-CoV-2	CS	RT-PCR	1	2	+
33^(43)^	SARS-CoV-2	CS	RT-PCR	36	72	+

## DISCUSSION

Our literature search found that viruses can be identified on the ocular surface through analysis of conjunctival swabs or Schirmer strips. However, most studies have focused on AdV, HSV, and SARS-CoV-2, and other viruses have not been thoroughly investigated.

Studies focused on the detection of AdV and HSV were linearly developed over the last 20 years and randomly conducted all over the world. In contrast, in 2020, scientists exponentially published studies on SARS-CoV-2 because the virus was first seen in the last days of 2019. During the first few months of 2020, the WHO declared it a pandemic, and the world’s research efforts were directed to overcoming this pandemic.

In contrast to SARS-CoV^(52)^, SARS-CoV-2 RNA was found by RT-PCR in conjunctival swabs^(38,39,40,41,42,43,44,45,46)^. However, samples were collected from a few patients and only a low and varying percentage presented positive results and/or ocular symptoms^(44)^. Most of these studies were developed in China^(38,39,41,45)^, mainly because Chian was the first epicenter of the virus. In some cases, ocular manifestations are one of the first symptoms of COVID-19. As a result, some researchers believe that the eye may contribute to the understanding of COVID-19 pathophysiology^(4,5)^. Recent studies have proved the presence of angiotensin-converting enzyme 2 (ACE2) and transmembrane serine protease 2 (TMPSS2) in the conjunctiva, limbus, and cornea, with prominent staining in the superficial epithelium surface, which are key factors for SARS-CoV-2 infection in human cells^(53)^. These results support the necessity of ocular protection in preventing the spread of viruses.

Research on viral screening is, in most cases, correlated with external ocular symptoms with the purpose of solving clinical doubts about pathogenic agents and identifying methods for fast and accurate viral diagnosis. Studies on VZV, CMV, and EBV were mostly combined with HSV to determine coinfection and differential diagnosis^(30,31,32)^.

In contrast, only one study correlated viral detection on the ocular surface with intraocular symptoms. This study was published in 2000 and aimed to verify the efficacy of intravenous ganciclovir treatment in immunocompromised acquired immunodeficiency syndrome (AIDS) patients with CMV retinitis. The results showed high clinical relevance for confirming and differentiating diagnoses of CMV retinitis when ophthalmoscopic findings were determined by PCR methods of conjunctival swab samples^(47)^. No other studies on this topic were identified by this review, likely because the incidence of CMV retinitis in the AIDS population significantly decreased with the introduction of effective antiretroviral therapy and early accurate diagnosis^(54,55,56,57)^.

The most commonly used method for sample collection was conjunctival swabs. This method also collects conjunctival cells, while Schirmer strips only allow the collection of tears since fluids pass to the filter paper because of gravity, viscosity, and capillary flow dynamics -the same physical processes that explain how liquids impregnate porous materials differently^(58,59)^. Consequently, the collected samples represent two different materials: (1) tears, cells, and fluids dispersed in the conjunctival sac and tears and (2) substances dissolved in it.

In the identified studies, samples were typically analyzed by PCR or RT-PCR depending on the viral genetic material of DNA or RNA, respectively. PCR methods are widely used because they allow for the replication and detection of low loads of viral DNA/RNA^(60)^. The point-of-care test was also compared with PCR effectiveness, sensitivity, and specificity were high; however, this was only explored for AdV(^28)^, likely due to epidemiological factors related to uncontrolled and fast spread of AdV conjunctivitis(^61)^.

This review included 36 articles from diverse countries and time periods, which suggests worldwide interest in the detection of viruses on the ocular surface in the last decades. The findings of this review may contribute to future research by clarifying key concepts to support the design of future research on ocular viruses.

Lastly, this review is subject to several limitations. First, only three databases were consulted. New articles on the theme are constantly being published, and so relevant studies may have been missed. Moreover, the analyzed studies typically involved a small number of patients and lacked clear definitions of collection time and viral persistence since disease onset. Additional studies with larger populations and time data are necessary to develop more definitive conclusions on this issue.

In conclusion, viruses can be detected through the analysis of samples collected by Schirmer strips and conjunctival swabs. Prior studies were generally conducted to understand viral infection, to develop accurate diagnostic methods, and to follow patients’ responses to treatment.
